# Associations between Oxytocin Receptor (*OXTR*) Genotype and Elementary School Children’s Likability, Dis-likability and Friendship among Classroom Peers: A Longitudinal Study

**DOI:** 10.1007/s10964-018-0855-0

**Published:** 2018-04-27

**Authors:** Jin He, J. Marieke Buil, Hans M. Koot, Pol A. C. van Lier

**Affiliations:** 1 Department of Clinical, Neuro and Developmental Psychology, Vrije Universiteit Amsterdam, Van der Boechorststraat 1, Amsterdam, 1081 BT The Netherlands; 2 Amsterdam Public Health Research Institute, Van der Boechorststraat 7, Amsterdam, 1081 BT The Netherlands

**Keywords:** Oxytocin receptor gene, Rs53576, Children, Peer relationships, Longitudinal design

## Abstract

The single nucleotide polymorphism rs53576 of the oxytocin receptor (*OXTR*) gene is involved in forming and maintaining relationships in various social contexts. However, this has not been studied in the childhood peer context. The present study followed 359 children (51.6% girls) from age 9 to 12 to explore associations between *OXTR* rs53576 genotype (i.e., AA, AG or GG genotype) and three indicators of children’s relationships with peers: likability and dis-likability among, and friendship with, classroom peers. Our results showed that *OXTR* rs53576 was associated with likability among boys, but not with dis-likability and friendship or among girls. Boys with an A and a G allele (i.e., AG genotype) became increasingly more liked by their peers across the four-year studied period than those with two A alleles or two G alleles (i.e., AA and GG genotype). This study indicates that *OXTR* rs53576 genotype might influence children’s peer relationships, particularly their likeability among peers. Associations between *OXTR* rs53576 and peer relationships may differ depending on children’s sex and the specific type of peer-relationship under scrutiny.

## Introduction

Children’s relationships with their classroom peers play an important role in their social, emotional and behavioral development during elementary school years (Brendgen et al. 2002; Choukas-Bradley and Prinstein 2014). For example, children who are not liked by their peers or who have no or only a few friends are at risk of developing various social and psychological problems, such as becoming bullying victims (Hodges et al. 1999; van Lier and Koot 2010), and developing emotional and behavioral problems (Fontaine et al. 2009; Kiesner 2002; Ladd and Troop-Gordon 2003). Previous studies have indicated that a significant part of the differences in children’s peer relationships might be explained by their genetic makeup (Boivin et al. 2013; Brendgen et al. 2009). One of the genes implicated in forming and maintaining relationships with others is the single nucleotide polymorphism rs53576 of the oxytocin receptor (*OXTR*) gene. The goal of the present study is to investigate the potential association between *OXTR* rs53576 and children’s relationships with classroom peers. Specifically, we studied the association between *OXTR* rs53576 and children’s likability, dis-likability, and friendship as perceived by their classmates from ages 9 to 12 years.

The *OXTR* gene has been linked to social behaviors that are important for forming and maintaining social relationships (e.g., Kogan et al. 2011; Walum et al. 2012). One of the most studied polymorphisms within the *OXTR* gene is the single nucleotide polymorphism (SNP) rs53576 (Bakermans-Kranenburg and van IJzendoorn 2014; Li et al. 2015). Evidence from a wide range of studies, which together cover the developmental period from infancy to adulthood, has shown that this SNP is linked to factors important for building and maintaining social relationships, such as the cognitive capability to efficiently process social and emotional information (Choi et al. 2017; Lucht et al. 2013; Tops et al. 2011). Furthermore, *OXTR* rs52576 has been associated with social relationships in a broad range of contexts, such as interactions within the family, romantic relationships, and interactions with strangers (Bakermans-Kranenburg and van IJzendoorn 2014; Li et al. 2015). However, knowledge about the potential role of *OXTR* rs52576 genotype in social relationships with peers during childhood is yet far from complete and several important questions are still unanswered.

### Association between ×*OXTR* rs53576 and Peer Relationships

Whether variation within *OXTR* rs52576 is associated with the development of children’s peer relationships is as of yet unclear. To the best of our knowledge, no previous studies have investigated the association between *OXTR* rs53576 and classroom peer relationships of elementary school children. Some studies of preschoolers and elementary school children have found that the rs53576 polymorphism was linked to several characteristics that may indirectly affect children’s peer relationships. For example, among three to five-year-old children, *OXTR* rs53576 was associated with helping and sharing behaviors (Wu and Su 2015), as well as with negativity and avoidance in the context of social interactions (Kryski et al. 2014). In ten-year-old children, the polymorphism was associated with the ability to recognizing human faces, which is important for social interactions (Slane et al. 2014). In addition, among adolescents, a study found that variations in *OXTR* rs53576 predicted different developmental patterns of loneliness across a five-year span (van Roekel et al. 2013). Thus, there is growing evidence indicated that *OXTR* rs53576 is linked to personal characteristics and behaviors that may affect children’s relationships with peers. Yet, studies that directly focus on the role of *OXTR* rs52576 in the peer-context are lacking.

### Function of *OXTR* rs53576 Genotypes

Despite the attention from many studies, it remains unclear which specific allele of the *OXTR* rs53576 might be beneficial for forming and maintaining positive social affiliations and which allele might be considered ‘risky’, in that it may hamper the development of positive social bonds. The SNP rs53576 within the *OXTR* gene consists of a nucleotide guanine (G) to adenine (A) change, resulting in three allelic combinations, or genotypes, namely: AA, AG, and GG (Gillath et al. 2008). Currently, there is no empirical evidence about how the three *OXTR* rs53576 genotypes are associated with oxytocin density and functioning of the oxytocinergic system (Gimpl and Fahrenholz, 2001; van Roekel et al. 2013). Because of the lack of biological evidence, previous studies have inferred its genotype functioning from neurological, neuroendocrine and the abovementioned behavioral studies. For example, studies have detected associations between variants of *OXTR* rs53576 and activities of some brain areas (e.g., amygdala and hypothalamus; Waller et al. 2016; Wang et al. 2014) and some hormones (e.g., cortisol; Chen et al. 2011; McQuaid et al. 2015). Nevertheless, without precise information about how the *OXTR* rs53576 genotypes are related to oxytocin density and functioning of the oxytocinergic system, it is unclear how the genotypes should be coded in quantitative genetic studies. Possibly as a result of this uncertainty, previous studies have used different schemes to code *OXTR* genotypes when investigating its association with social outcomes.

Previous studies have used three different schemes of coding the *OXTR* rs53576 genotypes. The first scheme grouped individuals with an A allele (i.e., AA and AG genotypes) together, and contrasted this AA/AG group with people carrying GG genotype. This A-dominant model is based on the assumption that when both the A and G allele are present, only the A allele would be expressed and affect phenotypes. This coding scheme has resulted in inconsistent findings. Some studies found that GG carriers showed more comforting and sharing behaviors (Wu and Su 2015), more trust behaviors (Kogan et al. 2011; Krueger et al. 2012), more social empathy (Gong et al. 2017; Rodrigues et al. 2009), and better cognitive capability in processing visual and auditory information during social interactions (Tops et al. 2011; Verhagen et al. 2014), compared to individuals with an AA/AG genotype. Other studies found that AA/AG carriers, and not the GG carriers, exhibited good emotion recognition skills (Lucht et al. 2009), showed decreasing loneliness over time (van Roekel et al. 2013), and experienced less separation anxiety (Costa et al. 2009).

In the second group of studies, individuals with a G allele (i.e., GG and AG genotypes) are compared with individuals without a G allele (i.e., the AA genotype). This G-dominant coding scheme implicates that when both the A and G alleles are present, only the G allele would be expressed and affect phenotypes. Studies that used this scheme have also yielded conflicting results. One study found that the AA genotype was associated with more negative emotionality (Kryski et al. 2014) compared to the AG/GG genotype. However, other studies found that AA carriers scored better (i.e., lower) in a social impairment test (Park et al. 2010; Slane et al. 2014) and showed better emotion recognition skills for both positive and negative emotions (Lucht et al. 2009) than GG/AG carriers.

The third genotype-coding scheme considered the three allelic combinations AA, AG, and GG as three different groups and that the frequency of one of the alleles (A or G allele for *OXTR* rs53576) would influence a particular phenotype. For instance, if the strength of the association between *OXTR* rs53576 GG genotype and peer relationship would be represented by a parameter γ, then the strength increases γ-fold for genotype AG and by 2γ-fold for genotype AA (Clarke et al. 2011). In other words, the association between allele frequency and a particular phenotype is linear in nature. Using this additive model, Lucht and colleagues (2009) found level differences in positive affect across the three genotypes. Specifically, the AA genotype carriers had the lowest levels of positive affect, GG carriers with the highest levels of positive affect, and AG carriers with in-between levels of positive affect. However, other studies using this approach found no relationship between the three genotypes and indicators of social affiliation (e.g., attachment, Chen and Johnson 2012), social cognition (e.g., face recognition memory; Skuse et al. 2014), and emotional reactions or behaviors in a social context (e.g., sharing and trust; Apicella et al. 2010; Tabak et al. 2014).

One drawback of previous studies is that one coding scheme – the heterotic model − has not been considered, whereas the link between *OXTR* rs53576 and social relationships might also be heterotic. Genetic heterosis occurs when individuals with a heterozygote of a specific genetic polymorphism (i.e., two different alleles; AG in the present study) show different levels of a phenotype than individuals with homozygotes (i.e., two identical alleles; AA and GG in the present study; Comings and MacMurray 2000). Although this coding model has, to our knowledge, not been used to study the *OXTR* gene in humans, a review of molecular heterosis revealed that genetic heterosis is common in humans and may be prevalent in up to 50% of all genotype-phenotype associations (Comings and MacMurray 2000). Without the certainty of the biological functioning of the *OXTR* rs53576 genotypes, a heterosis effect of *OXTR* rs53576 on social affiliations cannot be ruled out. Evidence of heterosis among human studies has been discovered for the catechol O-methyltransferase receptor (*COMT*) gene, the dopamine receptor D2 (*DRD2*) gene, and the serotonin transporter gene (*SLC6A4*; Costas et al. 2011; Coutinho et al. 2004; Gelernter et al. 1993; Gosso et al. 2008; Middeldorp et al. 2007). For example, one study found that infants with heterozygotic genotypes of the *COMT* polymorphism had higher levels of attachment security (Luijk et al. 2011), compared to their homozygotic counterparts. Moreover, Comings and MacMurray (2000) found that some of the heterotic genotype-phenotype associations were blurred when using inaccurate coding scheme. This might also explain why the results of *OXTR* rs53576 and its association with social-relationship phenotypes are inconsistent (e.g., Bakermans-Kranenburg and van IJzendoorn 2014; Li et al. 2015), because the link between *OXTR* rs53576 and social relationships might also be heterotic and were potentially blurred when the genotypes were not coded properly.

In the present study, we therefore explored the modeling rs53576 genotypes with the four different genotype-coding schemes (i.e., an A-dominant model, a G-dominant model, an additive model, and a heterotic model) to investigate which specific allele underlies the association between *OXTR* rs53576 and the three indicators of children’s peer relationships of interest.

### Sex Difference of the *OXTR*-Peer Relationship Association

Another question about effects of *OXTR* rs53576 is whether the potential associations between variants of *OXTR* rs53576 and children’s peer relationships may be sex-specific. Findings concerning sex differences in social outcomes linked to the SNP are inconsistent. Some previous studies found that the associations between *OXTR* polymorphism and, for example, prosocial behavior, were stronger among males, compared to females (e.g., Kogan et al. 2011). Similarly, another study found that the association between *OXTR* and trust behavior (Krueger et al. 2012) was significant only for males and not for females. In contrast, several other studies found significant *OXTR* effects for females but not for males, for example, on loneliness (van Roekel et al. 2013; van Roekel et al. 2013). In contrast to these findings, there are also studies that did not find significant differences between males and females (e.g., Lucht et al. 2013), while other studies, especially some studies in childhood, did not consider sex differences (e.g., Slane et al. 2014; Wu and Su 2015). Based on these findings, sex differences in the association between *OXTR* rs53576 and peer relationships cannot be ruled out and, therefore, were investigated in the present study.

## Current Study

The goal of the present study was to investigate whether *OXTR* rs53576 would be associated with children’s peer relationships in the context of their elementary school classroom. We included three indicators of peer relationships as the outcome: likability, dis-likability, and friendship as perceived by classroom peers. We tested the effects of the *OXTR* gene with four different genotype-coding models in order to explore whether the potential genotype-phenotype associations would be affected by the way that alleles were coded. In addition, possible sex differences in the *OXTR*-peer relationship associations were also examined for each genotype-coding scheme and for each peer relationship outcome. Given the lack of studies on the association of the *OXTR* gene with peer relationships and the mixed results of previous studies on social outcomes, we formulated no a priori hypotheses.

## Methods

### Participants

Data of the present study (359 children, 48.4% boys) came from two longitudinal studies assessing children’s social, emotional and behavioral development across the elementary school years. In the first project, schools in the west and east of the Netherlands were invited to participate, and the first 30 schools that accepted this invitation were included in the project. At the beginning of the first project, parental informed consent was obtained for 90% of the schools’ children who were invited (Menting et al. 2011). In the second project, 18 schools from the north and east of the Netherlands were recruited through municipal health services. Parental informed consent was obtained for almost all children (99.9%; Gooren et al. 2011). The projects were reviewed by the medical ethics boards of the Erasmus Medical Center Rotterdam and the Vrije Universiteit Medical Center Amsterdam. Children were assessed annually during their elementary school years. In the first and second grades, a classroom-based preventive intervention program targeting behavioral problems (either the Good Behavior Game; Dolan et al. 1989; Van der Sar and Goudswaard 2001; or the Promoting Alternative Thinking Strategies curriculum, Kusché and Greenberg 1994) was implemented. Schools were randomly assigned to the intervention or the control condition. We oversampled children in the intervention condition so that approximately 60% of the children were in the intervention condition. After the second grade, all schools could decide whether or not to implement the intervention programs and the implementation was no longer monitored by the research team. Data from children in the third to sixth grade (from 9 to 12 years old, four assessments) were used in the present study. Given that the years included in the present study were out of the planned intervention period, we did not expect intervention effects on the studied associations. Nevertheless, possible intervention effects on all studied associations were tested.

All children who participated and remained in the final assessment of the longitudinal projects (*N* = 1091) were invited to provide a saliva sample for DNA extraction at age 13. Of those, a total of 406 children provided saliva samples and *OXTR* rs53576 genotype was subtracted successfully for 400 children. Among them, 41 children had no peer relationship data on the waves included in the present study (the 3rd–6th grade), and hence were not included. As a result, data from a total of 359 children (48.4% boys) were included in the analyses. Of these children, 40 children had an AA genotype (21 boys; 52.5%), 164 had an AG genotype (83 boys; 50.6%) and 155 had a GG genotype (70 boys; 45.2%). The distribution of genotypes in the present sample was equal to the Hardy-Weinberg Equilibrium expectations (χ^2^(2) = .02, *p* = .99) in the European population. The majority of the participants (90.6%) were of the Dutch/Caucasian descent. Two point four percent (2.4%) of the included children were Moroccan; 1.2% Surinamese; 0.9% Turkish; and 2.1% of children belonged to other ethnic groups; 2.8 % had missing data on ethnicity. Of all children who were invited to provide saliva samples, children who did not consent to saliva collection did not differ from those who did on friendship (*F* (1, 820) = 0.31, *p* = .58), but scored higher on peer likability (*F* (1, 820) = 6.40, *p* = .01) and lower on peer dis-likability (*F* (1, 820) = 14.17, *p* < .001). Among children who provided a saliva sample, those who did not have peer relationship data on the waves included in the present study (*N* *=* 41) did not significantly differ on *OXTR* genotype distribution (χ^2^(2) = .46, *p* = .80), sex (χ^2^(1) = .11, *p* = .74), or intervention condition (χ^2^(1) = .41, *p* = .52) compared to children who had peer relationship data.

### Procedure

Schools were contacted through phone calls. The purpose and procedure of the projects were explained to the school principles. After the school principals granted permission, a letter that explained the procedures and measurements and a consent form were sent to the children’s parents. Only children with written parental consent participated in the study. Peer relationship data of the present study were collected during regular classroom hours in the classroom. Children were arranged to sit separately in order to assure privacy and were instructed not to talk with their classmates about the questionnaires. After the general instruction, children received a list with the names of all classmates who were granted consent to participate in the study. Children were instructed to nominate classmates (by checking classmates’ names) that fitted the description provided on top of the paper (e.g., who do you like most). After the measurements, children were offered a small gift as a token for their participation.

Written consent forms for the DNA study were sent through post to the parents of the children, and the children themselves if they were older than 12 years old. Saliva samples were collected using the Oragene^TM^ DNA Self-Collection Kit (OG-500 tube) according to the instruction of the manufacturer (DNAGenotek, Ottawa, Ontario, CAN). The self-collection kit consists of a tube in which children have to spit 2 ml of saliva (until the ‘fill line’ on the tube). The children were instructed not to eat, drink (except for water), smoke or chew gum 30 min before the saliva collection. Detailed instructions can be found at the manufacturer’s website (http://www.dnagenotek.com/ROW/pdf/PD-BR-017.pdf). Trained research assistants assisted the participants with collecting saliva at the participants’ homes and ensured that the children followed the instructions.

### Measures

#### *OXTR* rs53576 genotypes

*OXTR* rs53576 genotypes were subtracted from saliva samples. The polymorphism of *OXTR* rs53576 was genotyped using PCR and fragment analysis on a 3130 Genetic Analyzer (Life Technologies, Carlsbad, CA). Coding of genotypes varied according to the schemes: in the A-dominant model, AA and AG were combined and coded as 0, while GG was coded as 1; in the G-dominant model, AA was coded as 0, while AG and GG were combined and coded as 1; in the additive model, AA was coded as −1, AG as 0 and GG as 1; and in the heterotic model, AA and GG were combined and coded as 0, while AG was coded as 1.

#### Peer likability and peer dis-likability

Peer likability and dis-likability were assessed annually based on the protocol described by Coie and Kupersmidt (1983). Children were asked to nominate an unlimited number of classmates who they liked most and classmates who they liked least. For each child, the ‘liked most’ and ‘liked least’ nominations were divided by the number of children who participated in the nomination procedure from the same classroom minus one (children were not allowed to nominate themselves). This resulted in scores ranging from 0 to 1. For likability, higher scores indicate being liked more. For dis-likability, higher scores indicate being disliked more by peers.

#### Friendship

Friendship was assessed annually by asking children to nominate an unlimited number of classmates whom they considered as their friends. Because our focus was on how the target child was perceived by their classmates, unidirectional friendship nominations (i.e., a classmate nominated the target child as a friend, but the target child did not necessarily nominate this particular classmate back as a friend) were used in the analyses. The nominations each child received were divided by the number of children who participated in the nomination procedure minus one, resulting in scores ranging from 0 to 1. Higher scores indicate that the child was perceived as a friend by more classmates.

#### Children’s sex

Children’s sex was dummy coded as 0 (girls) and 1 (boys).

#### Principal coordinates for population stratification

Three constructs were included to control for possible confounding effects of population stratification on the effect of *OXTR* genotype. Children’s ethnicity represented approximately the global level of population stratification, coded as 1 (Dutch and Western immigrants, 90.6% of the sample) and 0 (other ethnicities). The local level of population stratification were represented approximately by two positional variables (North/South and East/West) of the children’s residential location according to a genome-wide study about the population structure in the Netherlands (Abdellaoui et al. 2013). The residences of children who participated in the present study were in three areas and were dummy coded as 1 (North) and 0 (South) for the North/South dimension and 1 (East) and 0 (West) for the East/West dimension.

#### Intervention status of children

Intervention status at first and second grade was dummy coded as 0 (control) and 1 (intervention).

#### Household socioeconomic status

Household socioeconomic status (SES) was assessed through children’s parental occupational status based on the Dutch Working Population Classification of Occupations Scheme (Statistics Netherlands 2017). The highest occupational level of the parent(s) was chosen to represent children’s household SES. Household SES was coded as 1 (unemployed to lower SES) and 0 (medium to higher SES) for each child.

### Statistical Analyses

**Fig. 1:**
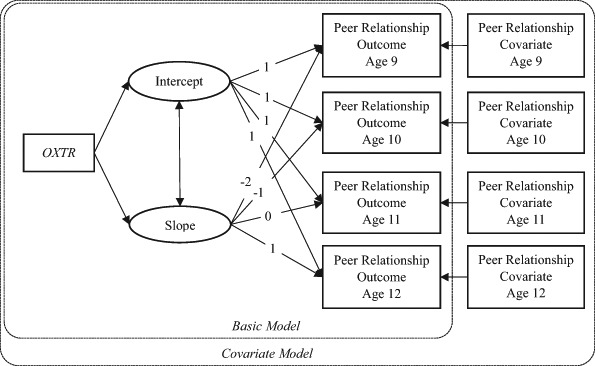
Conceptual model of *OXTR* (rs53576) genotype as a predictor of the peer relationship outcome variable (i.e., likability, dis-liability or friendship) without (*Basic Model*) or with (*Covariate Model*) one of the other two peer relationship covariates added as time-varying covariates from age 9 through 12

Associations between *OXTR* rs53576 genotypes and peer relationships were estimated through latent growth curve models (LGCM, Muthén and Khoo 1998; see Fig.1). LGCMs were fitted separately for the three peer relationship outcomes (i.e., peer likability, peer dis-likability, and friendship). LGCMs estimate two growth parameters for each outcome: the level of the peer relationship outcome, represented by an *intercept*, and the developmental trend of the outcome across the whole studied period (i.e., from age 9 to 12 years), represented by a linear *slope*. The intercept was centered at the age of 11 years to estimate the level of peer relationships in the middle of the covered period. Paths from *OXTR* rs53576 genotype to both parameters were modeled to represent *OXTR*-peer relationship associations.

The analyses were performed in two steps. In the first step, we used model fit indices of the LGCMs to select the genotype-coding model that fitted the best for *OXTR*-peer relationships associations and to estimate sex differences in the associations. To estimate the best fit genotype-coding model, we compared the model fit indices that are allowed for non-nested model comparisons (Curran et al. 2010; Widaman and Thompson 2003) of models with *OXTR* genotype being coded according to four different models: the A-dominant model, the G-dominant model, the additive model, and the heterotic model. The model fit indices for non-nested model comparisons included Akaike’s information criterion (AIC; Akaike 1998), the Bayesian information criterion (BIC; Schwarz 1978), and the sample-size adjusted Bayesian information criterion (aBIC; Sclove 1987). For all three model-fit indices, smaller values indicate better fit. To estimate sex differences in the *OXTR*-peer relationship associations, the LGCMs were fitted with multiple group comparisons (boys vs. girls), in which the fit of a model with *sex-specific* estimates was compared to the fit of a model in which effects were held equal across sex (*sex-equal* model). Changes in chi-squares of the two models were tested using the Satorra-Bentler test (SB test, Satorra 2000), with a significant change indicating sex differences.

After the best fit genotype-coding model and sex model were selected, we performed the second step of our analysis, in which the associations between *OXTR* genotypes and the peer relationship outcomes were estimated. The LGCMs were estimated for each peer relationship outcome separately. The association between *OXTR* rs53576 genotypes and the peer relationship outcomes (i.e., either likability, dis-likability or friendship) was estimated first without covariates (*basic* model), then with the covariance between the target outcome and other indicators of peer relationship, by including one of the other two indicators of peer relationship as a time-varying covariate (*covariate model*; see Fig. 1).

Children’s household socioeconomic status, intervention status, and the principal coordinates for population stratification (i.e., ethnicity and area of residence) were controlled as predictors of the phenotype growth parameters in all latent growth curve models. Moreover, intervention status was also tested as a moderator on all genotype-phenotype associations, to see whether the intervention affected the studied associations. Models were fitted using the software Mplus 7.31 (Muthén and Muthén 1998–2015). Standard errors of all paths were adjusted for clustering of data within classrooms using a sandwich estimator (Williams 2000). Maximum likelihood estimation with robust standard errors (MLR) was used in order to account for the non-normal distributions of dis-likability and friendship. Missing data were handled with the full information maximum likelihood estimation (FIML).

## Results

### Preliminary Analyses

Descriptive statistics are reported in Table 1. Repeated ANOVAs showed that boys scored lower than girls on peer likability but higher on peer dis-likability. No sex difference for level of friendship was found. All correlations (see Table 2) between the studied variables were in the expected direction. Furthermore, the slope parameters of peer likability (slope = .02, *SE* = .01, *p* = .22), peer dis-likability (slope = −.01, *SE* < .01, *p* = .06), and friendship (slope = .01, *SE* < .01, *p* = .18) indicate that on average, the number of peer-nominations that children received for likability, dis-likability and friendship were stable from 9 to 12 years of age.

**Table 1:** Means and standard deviations of peer likability, peer dis-likability, and friendship across time

		Total sample		Boys		Girls		Sex differences *F* (*df*)
		*M*	*SD*	*M*	*SD*	*M*	*SD*	
Peer likability	T1	.37	.18	.35	.16	.39	.20	7.06 (1)**
	T2	.38	.18	.35	.17	.40	.19	
	T3	.37	.17	.34	.16	.40	.16	
	T4	.42	.18	.40	.18	.44	.19	
Peer dis-likability	T1	.18	.16	.21	.18	.15	.14	7.29 (1)**
	T2	.19	.16	.22	.17	.16	.15	
	T3	.17	.16	.20	.17	.15	.14	
	T4	.15	.15	.17	.17	.13	.13	
Friendship	T1	.25	.16	.25 .	25 .	26 .	18	2.74 (1)
	T2	.30	.16	.28	.28	.31	.18	
	T3	.29	.14	.28	.28	.30	.16	
	T4	.29	.16	.28	.28	.29	.17	

**p < .01

**Table 2:** Correlations between peer likability, peer dis-likability, and friendship

		1	2	3	4	5	6	7	8	9	10	11
Peer likability												
1	T1	–										
2	T2	.60	–									
3	T3	.42	.62	–								
4	T4	.39	.44	.60	–							
Peer dis-likability												
5	T1	−.48	−.47	−.38	−.26	–						
6	T2	−.34	−.50	−.43	−.35	.65	–					
7	T3	−.29	−.39	−.57	−.48	.51	.61	–				
8	T4	−.30	−.34	−.50	−.65	.47	.50	.67	–			
Friendship												
9	T1	.79	.54	.39	.37	−.35	−.21	−.20	−.18	–		
10	T2	.52	.77	.55	.41	−.44	−.43	−.40	−.37	.53	–	
11	T3	.29	.50	.78	.50	−.19	−.28	−.43	−.33	.28	.48	–
12	T4	.35	.38	.56	.79	−.16	−.26	−.39	−.49	.38	.42	.52

All correlations in the table are significant (*p* < .001)

### Associations Between *OXTR* rs53576 and Peer Likability, Dis-likability and Friendship

#### Peer likability

We first estimated models for the association between *OXTR* rs53576 and peer likability. Model fit results showed that the sex-specific heterotic model had the best fit according to the AIC and aBIC fit indices, while the sex-equal A-dominant model showed the best fit according to BIC (in Table 3). However, the SB test showed a significant sex difference for the A-dominant model. Therefore, we rejected the sex-equal A-dominant model and select the sex-specific heterotic model as the best fit model. We then tested intervention effects by adding intervention status as a moderator on the association between *OXTR* genotype and peer likability. The three-way interaction (i.e., sex × intervention status × *OXTR* genotype) was not significant (intercept: *B* = −.01, *SE* = .06, *p* = .86; slope: *B* = −.03, *SE* = .02, *p* = .23). Thus, invention status was then controlled by including it in the models as a covariate in the prediction of the growth parameters of likability.

**Table 3:** Model fit indicators of the four genotype-coding models for peer likability, peer dis-likability and friendship and sex difference tests

	Additive	A-dominant	G-dominant	Heterotic
Peer likability				
Sex-specific				
χ^2^	88.22	88.38	89.37	**89.30**
*df*	50	50	50	**50**
AIC	−852.05	−858.74	−848.39	**−861.18**
BIC	−697.16	−703.85	−693.51	**−706.30**
aBIC	−824.06	−830.75	−820.41	**−833.20**
Sex-equal				
χ^2^	91.77	96.61	92.46	100.70
*df*	52	52	52	52
AIC	−853.68	−854.87	−849.88	−852.91
BIC	−706.54	−707.73	−702.74	−705.77
aBIC	−827.09	−828.28	−823.29	−826.32
Sex specific vs. sex-equal model				
Δχ^2^	3.57	23.32^***^	2.80	40.69^***^
Peer dis-likability				
Sex-specific				
χ^2^	115.01	115.89	113.32	**115.55**
*df*	50	50	50	**50**
AIC	−1243.38	−1244.17	−1246.02	**−1247.03**
BIC	−1088.50	−1089.28	−1091.13	**−1092.14**
aBIC	−1215.40	−1216.18	−1218.03	**−1219.04**
Sex-equal				
χ^2^	120.68	121.66	120.68	122.86
*df*	52	52	52	52
AIC	−1243.42	−1243.87	−1243.96	−1244.74
BIC	−1096.27	−1096.73	−1096.82	−1097.60
aBIC	−1216.83	−1217.28	−1217.37	−1218.15
Sex specific vs. sex-equal model				
Δχ^2^	6.68^*^	6.64^*^	9.99^**^	9.04^**^
Friendship				
Sex-specific				
χ^2^	97.99	99.52	99.96	98.76
df	50	50	50	50
AIC	−1093.70	−1098.39	−1091.53	−1100.58
BIC	−938.82	−943.50	−936.64	−945.70
aBIC	−1065.72	−1070.40	−1063.54	−1072.59
Sex-equal				
χ^2^	99.72	**103.48**	98.09	105.39
*df*	52	**52**	52	52
AIC	-1097.35	**−1100.17**	−1091.92	−1098.48
BIC	-950.21	**−953.03**	−944.78	−951.34
aBIC	-1070.76	**−1073.59**	−1065.33	−1071.89
Sex specific vs. sex-equal model				
Δχ^2^	0.41	3.92	3.01	9.94^**^

Δχ^2^ statistics are based on the Satorra-Bentler Chi square difference test. The genotype-coding model chosen for each peer relationship indicator are marked bold

^*^p < .05, ^**^p < .01, ^***^p < .001

The model estimates (see Table 4) show that in the basic model, the associations between genotype and the intercept, as well as the slope of likability, was significant for boys, but not for girls. Specifically, boys with an AG genotype had higher level (i.e., intercept) and a significant increase (i.e., slope) of likability during the length of the study compared to boys with either an AA or a GG genotype. No association between *OXTR* genotype and likability was found for girls. When peer dis-likability was added to the model as a time-varying covariate, the association between *OXTR* and the growth parameters remained significant. However, when friendship was added as a covariate, the association between *OXTR* genotype and the intercept of peer likeability was no longer significant, while the association with the slope of peer likability remained significant.

**Table 4:** Association between *OXTR* genotypes and peer likability controlled for potential confounders

	Intercept				Slope			
	*B*	*SE*	β	*p*	*B*	*SE*	β	*p*
Basic model								
Boy								
SES	−.05	.03	−.14	.06	<.01	.01	−.03	.83
Intervention	.07	.03	.26	.01	−.02	.01	−.20	.83
Ethnicity	−.08	.05	−.18	.09	−.01	.01	−.11	.31
North/South	−.04	.06	−.13	.56	<.01	.03	−.01	.99
East/West	.02	.06	.08	.70	−.02	.03	−.32	.47
*OXTR* (heterotic)	.06	.02	.23	.01	.03	.01	.39	.001
Girl								
SES	−.03	.04	−.10	.39	−.03	.02	−.24	.16
Intervention	.05	.30	.20	.08	<.01	.02	−.01	.96
Ethnicity	−.08	.05	−.18	.10	−.05	.04	−.26	.25
North/South	−.09	.03	−.31	.01	−.06	.03	−.47	.07
East/West	<.01	.03	−.01	.95	−.04	.03	−.34	.18
*OXTR* (heterotic)	<.01	.02	.01	.88	−.02	.01	−.17	.13
Controlled for dis-likability								
Boy								
SES	−.02	.02	−.09	.28	.01	.01	.10	.67
Intervention	.05	.02	.30	.03	−.01	.01	−.25	.40
Ethnicity	−.10	.03	−.33	.002	−.02	.01	−.25	.22
North/South	−.04	.05	−.21	.43	−.01	.02	−.11	.84
East/West	.01	.05	.07	.78	−.02	.02	−.54	.31
*OXTR* (heterotic)	.03	.01	.17	.03	.02	.01	.44	.004
Girl								
SES	.01	.03	.05	.50	−.02	.02	−.19	.42
Intervention	.04	.03	.20	.70	.01	.01	.11	.50
Ethnicity	−.07	.04	−.22	.10	−.02	.04	−.16	.54
North/South	−.05	.04	−.23	.19	−.05	.03	−.60	.04
East/West	.02	.03	.10	.56	−.04	.02	−.48	.05
*OXTR* (heterotic)	.01	.02	.03	.75	−.02	.01	−.17	.15
Controlled for friendship								
Boy								
SES	−.03	.02	−.19	.30	−.01	.01	−.17	.43
Intervention	.02	.01	.15	.01	−.01	.01	−.45	.13
Ethnicity	<.01	.03	.01	.93	−.01	.01	−.18	.38
North/South	−.02	.02	−.17	.35	.01	.01	.20	.56
East/West	.04	.02	.33	.03	.02	.01	.58	.11
*OXTR* (heterotic)	.02	.01	.14	.09	.02	<.01	.51	.04
Girl								
SES	−.03	.01	−.25	.10	−.01	.01	−.22	.25
Intervention	.03	.02	.26	.02	<−.01	.01	−.02	.92
Ethnicity	.04	.03	−.22	.12	−.02	.03	−.26	.47
North/South	−.06	.02	−.57	.01	−.02	.02	−.38	.21
East/West	<−.01	.02	−.01	.98	−.02	.01	−.35	.11
*OXTR* (heterotic)	<.01	.01	.03	.72	−.01	<.01	−.20	.12

#### Peer dis-likability

Model fit results (see Table 3) showed that the sex-specific heterotic model fitted the data best according to AIC and aBIC values, while the sex-equal heterotic model showed the best fit according to BIC. However, SB test showed significant sex differences for all genetic models. Thus, the sex-specific heterotic model was chosen as the best fit model. Again, we tested for potential intervention effects. The three-way interaction (i.e., sex × intervention status × *OXTR* genotype) was not significant (intercept: *B* = .04, *SE* = .05, *p* = .48; slope: *B* < .01, *SE* = .02, *p* = .93). Invention status was therefore controlled by including it in the models as a covariate.

Results in Table 5 show a significant effect of genotype on the intercept of peer dis-likability for boys only in the basic model. No significant association between *OXTR* genotype and the slope of dis-likability was found. Also, no association between *OXTR* genotype and dis-likability (intercept or slope) was found for girls. Moreover, after including peer likability or friendship as time-varying covariates, the association between peer dis-likability and *OXTR* genotype was no longer statistically significant, indicating that the *OXTR-*peer dis-likability association might be explained by the associations between the genotype and peer likeability or friendship.

**Table 5:** Association between *OXTR* genotypes and peer dis-likability controlled for potential confounders

	Intercept				Slope			
	*B*	*SE*	β	*p*	*B*	*SE*	β	*p*
Basic mode1								
Boy								
SES	.06	.04	.06	.13	.01	.01	.18	.21
Intervention	−.03	.02	−.12	.14	.02	.02	.08	.55
Ethnicity	−.03	.05	−.05	.60	−.01	.02	−.02	.86
North/South	−.02	.04	.02	.91	−.02	.03	−.20	.50
East/West	<.01	.04	−.05	.70	<.01	.02	<.01	.99
*OXTR* (heterotic)	−.05	.03	−.20	.03	−.01	.10	−.16	.18
Girl								
SES	.08	.03	.32	.04	.02	.01	.14	.06
Intervention	−.03	.02	−.14	.16	.02	.01	.17	.04
Ethnicity	.10	.04	.03	.79	.05	.02	.23	.01
North/South	.07	.02	.34	.001	−.01	.02	.23	.43
East/West	.04	.02	.20	.02	−.02	.02	.25	30
*OXTR* (heterotic)	<.01	.01	.01	.84	.01	.01	.09	.44
Controlled for likability								
Boy								
SES	.03	.03	.13	.28	.01	.01	.05	.80
Intervention	.01	.02	.01	.97	.01	.02	.21	.28
Ethnicity	−.07	.03	−.20	.04	−.02	.02	−.15	.42
North/South	−.02	.03	−.08	.65	−.02	.01	−.38	.03
East/West	−.01	.03	−.03	.85	−.01	.01	−.20	.18
*OXTR* (heterotic)	−.02	.02	−.12	.14	<.01	.01	.04	.79
Girl								
SES	.07	.02	.37	.003	.01	.01	.16	.55
Intervention	−.01	.02	−.09	.47	.03	.01	.76	.01
Ethnicity	−.02	.03	−.06	.63	.03	.02	.54	.06
North/South	.04	.02	.27	.06	−.03	.01	−.90	.001
East/West	.04	.02	.26	.08	−.03	.01	−.78	.003
*OXTR* (heterotic)	<.01	.01	.02	.81	<.01	.01	<.01	.99
Controlled for friendship								
Boy								
SES	.05	.03	.02	.14	.01	.02	.01	.51
Intervention	−.01	.02	.03	.78	.02	.01	.02	.13
Ethnicity	−.07	.04	.04	.08	−.01	.02	.02	.64
North/South	−.01	.04	.04	.91	−.03	.01	.01	.03
East/West	−.03	.04	.04	.45	−.03	.01	.01	.02
*OXTR* (heterotic)	−.03	.02	−.14	.07	−.01	.01	−.08	.57
Girl								
SES	.08	.03	.37	.23	.01	.01	.25	.20
Intervention	−.02	.02	−.12	.002	.02	.01	.61	.02
Ethnicity	−.01	.04	−.01	.91	.04	.02	.58	.02
North/South	.06	.03	.34	.02	−.02	.01	−.57	.01
East/West	.04	.03	.22	.14	−.02	.01	−.56	.03
*OXTR* (heterotic)	<.01	.01	.02	.79	<.01	.01	.07	.56

#### Friendship

Model fit results (in Table 3) showed the that the sex-specific heterotic model was the best fitting model according to the AIC, but the BIC and aBIC indicated that the sex-equal A-dominant model had the best fit. SB test also showed conflicting results, with significant sex differences for the heterotic model, but no significant sex difference for A-dominant model. The sex-equal A-dominant model was preferred by two out of three model indices (i.e., BIC and aBIC) and the other (i.e., AIC) had a value very close to the best fit value (AIC of sex-specific heterotic model). Therefore, the sex-equal A-dominant model represented the *OXTR*-friendship association best. Testing for potential moderation by intervention yielded no significant results (intercept: *B* = −.03, *SE* = .03, *p* = .40; slope: *B* = −.01, *SE* = .01, *p* = .51). Sex and invention status were controlled as covariates in the model.

Results of the basic model in Table 6 show a significant association between *OXTR* genotype with the A-dominant model and the intercept of friendship, suggesting that children with an AA or AG genotype were more often nominated as a friend than those with a GG genotype. No significant association between *OXTR* genotype and the slope of friendship was found. After including peer likability in the model as a time-varying covariate, the association between genotype and the intercept of friendship became non-significant; whereas it remained significant when peer dis-likability was the covariate. Our findings indicate that the *OXTR-*friendship association overlaps with *OXTR*-peer likability association.

**Table 6:** Association between *OXTR* genotypes and friendship controlled for potential confounders

	Intercept				Slope			
	*B*	*SE*	β	*p*	*B*	*SE*	β	*p*
Basic model								
SES	−.01	.02	−.01	.96	−.01	.01	−.07	.63
Intervention	.04	.03	.19	.10	−.01	.01	−.11	.51
Sex	.03	.02	.12	.09	.01	.01	.06	.58
Ethnicity	−.06	.03	−.18	.05	−.01	.02	−.11	.45
North/South	−.02	.06	−.11	.69	−.01	.02	−.15	.64
East/West	−.01	.05	−.06	.82	−.03	.02	−.38	.21
*OXTR* (A-dominant)	−.03	.01	−.14	.02	<.01	.01	−.01	.95
Controlled for likability								
SES	.02	.01	.18	.07	.01	.01	.06	.81
Intervention	.01	.01	.02	.91	.01	.01	.21	.60
Sex	−.01	.01	−.15	.12	<.01	.01	.02	.96
Ethnicity	−.01	.02	−.10	.45	.01	.01	.15	.66
North/South	.02	.03	.25	.53	−.01	.01	−.32	.58
East/West	−.02	.03	−.21	.55	−.01	.01	−.75	.11
*OXTR* (A-dominant)	−.01	.01	−.07	.47	<.01	.01	.21	.42
Controlled for dis-likability								
SES	.02	.02	.36	.29	<−.01	.01	.02	.92
Intervention	.03	.02	.11	.19	<.01	.01	−.02	.96
Sex	<.01	.01	.02	.83	.01	.01	.08	.55
Ethnicity	−.07	.03	−.23	.02	−.01	.02	−.07	.72
North/South	−.01	.06	−.06	.86	−.02	.02	−.37	.32
East/West	−.01	.05	−.04	.90	−.03	.02	−.64	.06
*OXTR* (A-dominant)	−.02	.01	−.14	.03	<.01	.01	.05	.71

## Discussion

Previous studies have found that the single nucleotide polymorphism rs53576 of the oxytocin receptor (*OXTR*) gene was associated with various behavioral and social-cognitive characteristics that are important for forming and maintaining interpersonal relationships. However, the influence of this polymorphism on the development of peer relationships in childhood was unknown. Moreover, previous studies used different genotype coding schemes to investigate the influence of the three *OXTR* genotypes (i.e., AA, AG, and GG) on social relationships and found inconsistent results. Finally, whether the effect of *OXTR* rs53576 varies across sexes was unclear. Thus, the present study investigated the association of *OXTR* rs53576 with three indicators of children’s peer relationships – likability and dis-likability, and friendship – among 359 elementary school children followed annually from age 9 to 12 years. Moreover, we took all possible genotype coding models into account and investigated the effect of *OXTR* genotype on children’s peer relationships while taking into account potential overlap between the three peer relationship outcomes.

With this explorative approach, we found that the *OXTR* rs53576 polymorphism, in general, was associated with the development of children’s peer relationships. However, the specific associations differed across the peer relationship outcomes and sex. We found that the association between *OXTR* genotype and peer likability remained significant when the overlap with the other two peer relationship outcomes (i.e., dis-likability and friendship) was taken into account. However, the associations between *OXTR* genotype and dis-likability and between *OXTR* and friendship dropped from significant to non-significant after we controlled their covariance with likability. This indicates that the association between *OXTR* rs53576 genotype and children’s peer relationships is mainly driven by peer likability, instead of by dis-likability or friendship. Furthermore, we found that the association of *OXTR* genotype with the development of peer likability was significant only for boys, and not for girls. Specifically, boys with an AG genotype became increasingly more liked by classmates from age 9 to 12 years, compared to boys with AA or GG genotype.

Previous research has shown that *OXTR* genotype is associated with social behaviors within the family or in interaction with strangers (Feldman et al. 2012; Krueger et al. 2012). The present study extends our knowledge of *OXTR* genotype effect into context of classroom. Moreover, we found that the magnitude of the association between *OXTR* genotype and dis-likability, as well as the association between *OXTR* genotype and friendship, were weakened when the effect of likability was taken into account. This indicates that peer likability might be the main aspect of children’s peer relationship that is associated with *OXTR* rs53576. Some previous studies also investigated the association of OXTR genotypes with multiple social-relationship outcomes (e.g., Lucht et al. 2009; Chen and Johnson 2012). However, these studies did not consider the potential interconnections between them. Our findings indicate the importance of taking potential overlap between different phenotypes into account when investigating the role of *OXTR* rs53576.

Regarding the question about how the three *OXTR* rs53576 genotypes should be coded when investigating the association between *OXTR* rs53576 and peer relationship phenotypes, our results are inconclusive. We found support for the heterotic model when peer likability and dis-likability were the outcomes, while for friendship it was the A-dominant model that fitted the data best. However, no previous studies have considered the heterotic model when investigating the effect of *OXTR* rs53576 on the development of social relationships. Our findings warrant further investigation of the heterotic genotype-coding model when studying associations between *OXTR* and phenotypes. In addition, our finding that different coding schemes showed the best fit to the data dependent on which specific peer relationship was investigated also suggests that the effect of *OXTR* rs53576 on peer relationship might differ for specific aspects of relationship. Furthermore, we also found that association between *OXTR* genotype and friendship was weakened when likability was taken into account. This might suggest that the genotype-coding model of the *OXTR* rs53576 in association with likability (i.e., the heterotic model) might outweigh the model of this SNP in association with friendship. Therefore, the heterotic model might be the best genotype-coding model to describe the associations between *OXTR* genotype and children’s peer relationships. Our findings, although cannot provide a definite answer about the correct genotype-coding scheme for *OXTR* rs53576, suggest that more caution on genotype coding is required and that all schemes need to be considered in future investigations, especially given the lack of knowledge about *OXTR* polymorphism functioning.

Regarding the potential gender differences in *OXTR*-peer relationship associations, we found that the association of *OXTR* rs53576 with peer likability existed only for boys. This may be due to the different social norms or expectations that exist for boys and girls. Previous research has indicated that when both boys and girls showed higher oxytocin-related socio-emotional competence, such as empathy (Gong et al. 2017; Rodrigues et al. 2009). Empathetic boys might be more ‘visible’ to peers than empathetic girls because empathetic behavior is less expected from boys compared to girls (Rose and Rudolph 2006). As a result, boys might particularly benefit from a genotype that stimulates positive social relationships.

Several limitations should be noted when interpreting our findings. First, the present study investigated only one SNP of the *OXTR* gene. There is growing evidence that other SNPs of the *OXTR* gene and SNPs of other genes may be associated with social competence as well (DiLalla et al. 2015; Poulin et al. 2012). Future studies need to consider other SNPs or use a genome-wide approach to provide more comprehensive information about the genetic influence on children’s peer relationships. Second, it is uncertain whether our findings can be extended to a larger population. Although our sample was comprised of children from mainstream elementary schools, children from minority ethnicities (9.4%) were under-represented in the present study (the nationwide rate is around 20% among children aged from 9 to 12; Statistics Netherlands 2017). Those children who did not participate our longitudinal projects might also experience more problems in peer relationships, which might indicate selective attrition hence hampering generalizability. In addition, although we controlled for the potential effect of population stratification with three approximate constructs, we did not have enough SNPs to generate principal coordinates specific to our sample. Thus, we cannot completely exclude the effect of systematic ancestry differences in the genetic makeup of the children in our study. Lastly, the peer relationship phenotypes assessed in the present study were limited to those within children’s classroom context. Children might have relationships with peers from other classes or outside school, which were not covered by our study.

## Conclusion

Overall, the present study suggests that *OXTR* rs53576 genotype is associated with the development of peer likability in boys from age 9 to 12 years. Boys with an AG genotype became increasingly more liked among their peers compared to boys with other genotypes (i.e., AA or GG). Moreover, peer likability might be the main aspect of children’s peer relationship (relative to dis-likability and friendship) that might be associated with *OXTR* rs53576. Future studies should consider all potential genotype-coding models and the role of sex, as well as include multiple relationship phenotypes and the potential overlap between phenotypes, to provide more comprehensive knowledge about the effect of the *OXTR* gene on social relationships.
